# Biomechanical Analysis of a Growing Rod with Sliding Pedicle Screw System for Early-Onset Scoliosis

**DOI:** 10.1155/2019/9535070

**Published:** 2019-06-12

**Authors:** Zhihua Ouyang, Wenjun Wang, Nicholas Vaudreuil, Robert Tisherman, Yiguo Yan, Patrick Bosch, James Kang, Kevin Bell

**Affiliations:** ^1^Department of Spine Surgery, The First Affiliated Hospital of University of South China, Hengyang, Hunan, China; ^2^Ferguson Laboratory for Spine Research, Department of Orthopaedic Surgery, University of Pittsburgh School of Medicine, Pittsburgh, PA, USA; ^3^UPMC Children's Hospital of Pittsburgh, Pittsburgh, PA, USA; ^4^Thornhill Family Professor of Orthopaedic Surgery, Harvard Medical School, Boston, MA, USA

## Abstract

Early-onset scoliosis (EOS) remains a challenging condition for which current nonfusion surgeries require iterative lengthening surgeries. A growing rod with sliding pedicle screw system (GRSPSS) was developed to treat spinal deformities without repeated operative lengthening. This study was performed to evaluate whether GRSPSS had similar stability as a conventional pedicle screw system to maintain deformity correction. A serial-linkage robotic manipulator with a six-axis load cell positioned on the end-effector was utilized to evaluate the mechanical stability of the GRSPSS versus conventional fixed scoliosis instrumentation. Ten skeletally mature thoracic female Katahdin sheep spines (T4-L1) were subjected to 2.5 Nm of flexion-extension (FE), lateral bending (LB), and axial rotation (AR) in 2° increments for each state. The overall range of motion (ROM), apical segment ROM, and stiffness were calculated and reported. A two-tailed paired *t*-test was used to detect significant differences (*p* < 0.05) between the fixed group and GRSPSS fixation. There were no significant differences in overall range of motion (ROM), apical segment ROM, or stiffness for FE or LB between the GRSPSS group and fixed group. In AR, the GRSPSS group showed increased ROM compared to the fixed group for the overall spine (36.0° versus 19.2°, *p* < 0.01) and for the instrumented T8-T10 segments (7.0° versus 2.9°, *p*=0.02). Similarly, the fixed rod elastic zone (EZ) stiffness was significantly greater than the GRSPSS EZ stiffness (0.29 N/m versus 0.17 N/m, *p* < 0.001). The space around the rod allows for the increased AR observed with the GRSPSS fusion technique and is necessary for axial growth. The GRSPSS fusion model shows equivalent flexion and LB stability to current fusion models and represents a stable fusion technique and may allow for longitudinal growth during childhood.

## 1. Introduction

Early-onset scoliosis (EOS), defined as >10° of spinal curvature prior to age 10 years old, is a challenging condition for surgeons to treat due to the young age of onset and the vertical growth potential after initial diagnosis [1]. Conservative treatments, such as bracing or casting, are effective at preventing deformity progression in many cases [2]. In the past, surgical treatments for EOS involved anterior or posterior fusion surgeries with rigid spinal instrumentation with rods and fixed pedicle screws [3–5]. However, some studies have shown that traditional fusion surgeries for EOS result in limited growth, loss of movement, adjacent segment degeneration, and limited thoracic development causing severe restrictive lung disease [3–7].

Adequate deformity correction, achieving optimal spinal growth, allowing for lung development, and the high postoperative complication rate make satisfactory surgical treatment very challenging. As a result, nonfusion surgeries have gained popularity with surgeons [8]. Most current instrumentation techniques, without fusion, still require iterative lengthening surgeries as the patient ages, and are prone to complications such as infection, rod breakage, joint fusion, and screw pullout [9–13].

The primary nonfusion techniques include single or dual fixed growing rods, such as the Harrington and Luque systems, and vertical expandable prosthetic titanium rib (VEPTR) [14]. The construct typically utilizes two adjacent solid rods, the first attached at the most cranial portion of the curve requiring correction and the second attached to the most caudal portion, which overlaps in a tandem connector attachment. The point of overlap is the site at which serial open lengthenings are performed with iterative surgeries. The rods are not fixed at the apex of the curve. One of the primary limitations is that they require repeated lengthening surgeries and have moderate complication rates despite the relative advantages of each system [15].

Recently, magnetically controlled growing rods have been shown to provide satisfactory deformity correction and avoid repeated surgeries [16–19]. The magnetic system is constructed of rods locked at the cranial and distal portions of the curve without fixation at the apex. Growth is achieved through a lengthening mechanism in the rod, which is activated at specified increments in clinic, and these constructs are not intended to require repeat surgical intervention. In the largest multicenter study to date involving 30 patients, complication rates for magnetic rods were comparable to those found in fixed rods, resulting in repeated surgeries in 30% of patients and an overall complication rate of 80%, including 3/30 rod breakages, 6/30 failures of lengthening, and 3/30 wound complications or infections [20]. In addition, the study found that magnetic rods have a significant rate of distraction effectiveness [20].

To address these concerns, more recent constructs that allow guided growth have been developed that do not require iterative surgeries. Currently, there are two guided growth systems available—the SHILLA growth guidance system and the growing rod with sliding pedicle screw system (GRSPSS).

In the SHILLA system, fixed length solid rods are fused to pedicle screws at the apex of the deformity, while at the cranial and caudal ends, the rod is captured by sliding pedicle screws, which are not locked in place and therefore guide the rods as the child grows. The system allows for controlled growth to the extent that rod length is left available past the sliding pedicle screws. Clinical studies of the SHILLA system have shown promising results despite a high incidence of instrumentation complications [20–22].

The GRSPSS has a unique two-part (external and internal) locking mechanism that is designed to improve surgical efficiency by enabling a single polyaxial screw to have both sliding (external mechanism is tightened) and fixed (internal mechanism is tightened) capabilities. The GRSPSS system also uses sliding screws that function, first, to maintain coronal and sagittal correction, and second, to guide the growth of the spine during skeletal growth. The system's design allows for vertebral growth outside the fused apex in the cephalad and caudal directions. The authors have previously shown that this GRSPSS allowed continued cranial growth in an *in vivo* porcine model [16].

Wilke et al. recommended that new implants and surgical approaches initially be tested through calibrated *in vitro* methods for primary stabilization in the main anatomical directions, including flexion-extension (FE), lateral bending (LB), and axial rotation (AR) [17]. However, few documented *in vitro* biomechanical analyses of any other commercially available system for instrumentation without fusion have been published. Therefore, the objective of the present study was to analyze *in vitro* biomechanical properties of the GRSPSS system compared to a conventional, fixed rod instrumentation in an ovine model.

## 2. Methods

### 2.1. Device Description

**Figure 1:**
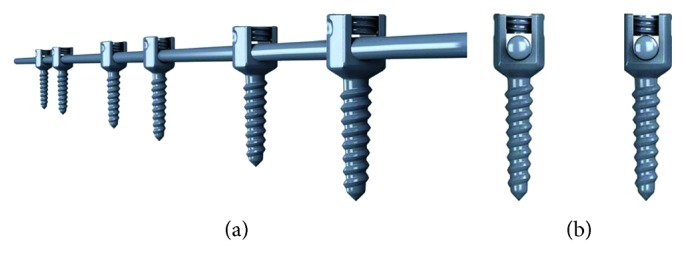
(a) Unilateral components of GRSPSS system including fixed screws in the apex and sliding screws in both ends. (b) Sliding pedicle screw (left) showing vertical gap, which allows for motion of the rod through the screw after it is fully tightened. Typical fixed pedicle screw (right).

The GRSPSS construct includes three parts: two 5.5 mm-diameter titanium rods, sliding pedicle screws, and conventional pedicle screws (Huasheng Inc., Changzhou, China). Each sliding pedicle screw consists of a head that uses a set screw to capture the rod without locking the rod in place. When the rod is secured beneath the set screw, there is a 0.75 mm distance between the top of the rod and the bottom of the set screw that permits the rod to slide in its groove in the longitudinal direction. Conventional pedicle screws, with set screws that lock the rod in place, were inserted at the apex of the spinal deformity. Finally, sliding screws were placed in the vertebrae at the cephalad and caudal ends of the specimen (Figure 1).

### 2.2. Specimen Preparation

Thoracic spine segments (T4-L1) from ten skeletally mature (>1 year old) female Katahdin sheep, previously shown to approximate the structure of human thoracic vertebrae [18], were acquired from a local abattoir for biomechanical testing and frozen at −20°C until testing. Spines were thawed at 4°C overnight and wrapped in saline-soaked gauze prior to testing. Once specimens were thawed, they were dissected of all paraspinal musculature and all ribs were transected 5 cm from the costovertebral joints. Transverse processes, all joint articulations, and all spinal ligaments remained intact. Specimens were visually inspected for the presence of large osteophytes, scoliosis, or other structural pathologies, and all specimens were found to be free of visual pathology. During testing, 0.9% saline was applied every 10–15 minutes to prevent desiccation.

**Figure 2:**
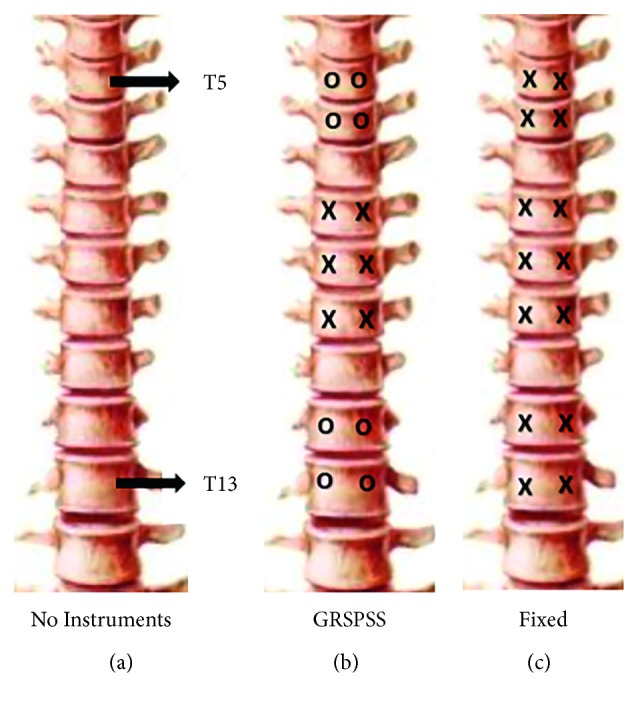
Diagram of experimental states showing no instrumentation (a), conventional fixed instrumentation (b) and the GRSPSS (c). × denotes traditional fixed pedicle screws; о denotes sliding pedicle screws.

Specimens were instrumented with seven pairs of thoracic pedicle screws (Huasheng Inc., Changzhou, China) at T5-6, T8-10, and T12-13. Three different states were investigated (Figure 2): no instrumentation group, GRSPSS group, and a fixed rod group, which used conventional thoracic fusion instrumentation with dual rigid rods (5.5 mm diameter titanium rods) locked in place with set screws. The GRSPSS group was instrumented bilaterally with conventional screws at T8-10 (locked apical fixation) and bilaterally with sliding screws in the upper (T5-6) and lower (T12-13) segments. Specimens from each group were mounted to the robotic testing fixture via custom fixation with pedicle screws cranially (T4) and caudally (L1) [19].

**Figure 3:**
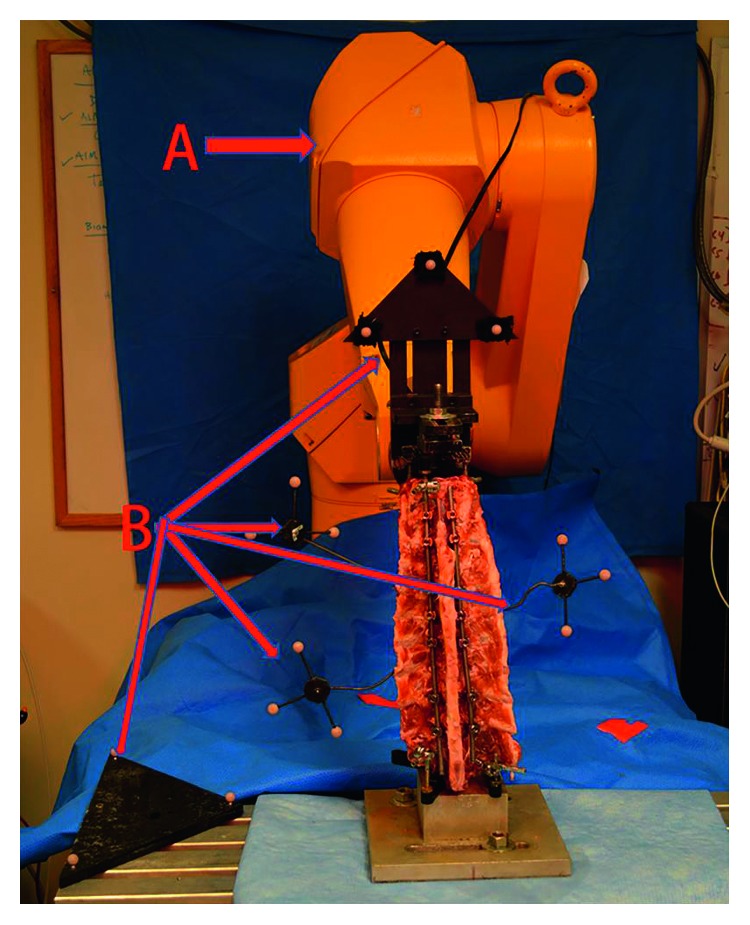
Image of the experimental setup: (A) serial-linkage robotic manipulator and (B) six-axis load cell attached to custom-built specimen mount on end-effector.

The robotic spine testing system consisted of a serial-linkage robotic manipulator (Staubli RX90, Stauli Inc., Duncan, SC), a six-axis load cell positioned on the end-effector (UFS Model 90M38A-150, JR3 Inc., Woodland, CA), and a custom-built specimen mounting fixture (Figure 3) [19]. The robot was controlled via a custom PC-based control algorithm written in MATLAB (R2012a, Mathworks Inc., Matick, MA). Five optical tracking cameras (M2, VICON Inc., Denver, CO) and motion tracking system (V460 Workstation, VICON Inc., Denver, CO) were used to track motion along the spine throughout testing. Motion of individual segments was tracked using reflective markers attached to T8, T9, and T10, representing the fused segment, as well as at the upper and lower mounting fixtures (rigidly attached to T4 and L1) of the robotic testing system. Prior to testing, three bony landmarks were recorded for the T4, T8-10, and L1 vertebrae to allow for accurate tracking of individual bones within their respective coordinate systems.

### 2.3. Experimental Protocol

The specimens from each group were subjected to 2.5 Nm of FE, LB, and AR in 2° increments at approximately 5 s/degree to simulate physiologic loading for each state [23]. Preliminary testing was performed to ensure that a moment target of 2.5 Nm was beyond the neutral zone (NZ) and within the elastic zone (EZ) of the s-shaped moment-rotation curve. The specimen groups underwent two cycles of preconditioning for each motion to minimize viscoelastic memory effects, and the motion of the third trial was used for all analyses [24]. Quasi-static robotic control was used to simulate an *in vivo* flexibility test by minimizing nonprimary moments and off-axis forces and updating the center of rotation for each step in the movement as previously described [19]. The primary outcome was the range of motion (ROM) in the desired plane of interest, i.e. FE, LB, and AR. The apical segment ROM and overall stiffness were also calculated and reported.

A custom MATLAB program was used for VICON data analysis and transformed the marker coordinate system to the bony coordinate system for each step during all motions, from which the relative position and Euler angles corresponding to LB, FE, and AR were determined in this conventional order. NZ and EZ parameters were determined by fitting a double sigmoidal function moment-rotation data to define NZ as the high compliance region demarcated by extrema of the second derivative, as described by Smit et al. [25]. NZ and EZ stiffness were defined as the inverse of the slope of a linear fit of the function in the NZ and EZ, respectively.

### 2.4. Statistics

A two-tailed paired *t*-test was used to detect significant differences (*p* < 0.05) in ROM and stiffness data between the fixed group and GRSPSS fixation. Statistical comparison to the intact spine was not performed as the differences are not clinically relevant, but the motion of the intact spine is displayed in all charts. All values in the text and graphs are presented as mean ± 95% confidence interval unless otherwise noted.

## 3. Results

### 3.1. Overall ROM

**Figure 4:**
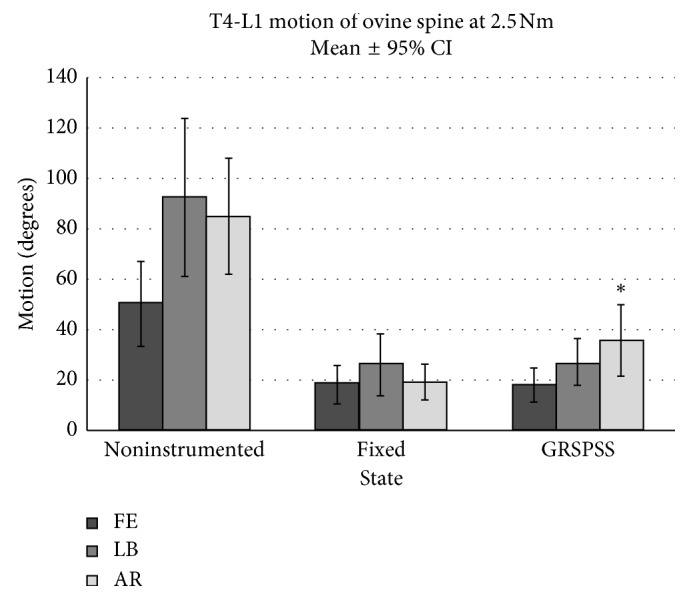
Overall ROM for all motions (FE, LB, and AR). Fixed and GRSPSS decreased motions significantly compared to no instrumentation. No significant difference between fixed and GRSPSS for FE or LB. GRSPSS had significantly more overall AR than fixed instrumentation.

For the specimens without instrumentation, the mean overall ROM in FE was 50.5° ± 16.8°, LB 92.8° ± 31.4°, and AR 85.1° ± 23.2° (Figure 4).

Between the GRSPSS and fixed rod states, no significant differences were observed in FE or LB. Overall ROM in FE was found to be 18.2° ± 7.4° for the fixed state and 18.0° ± 7.0° for the GRSPSS state (*p*=0.85). Similarly, LB ROM was 26.0° ± 12.6° for the fixed state and 27.2° ± 9.5° for the GRSPSS state (*p*=0.90).

The only significant difference found between the fixed and GRSPSS state in overall ROM was in AR. Fixed state AR was 19.2° ± 7.0° while the GRSPSS AR ROM was 36.0° ± 14.2° (*p*=0.008).

### 3.2. Apical Segment ROM

**Figure 5:**
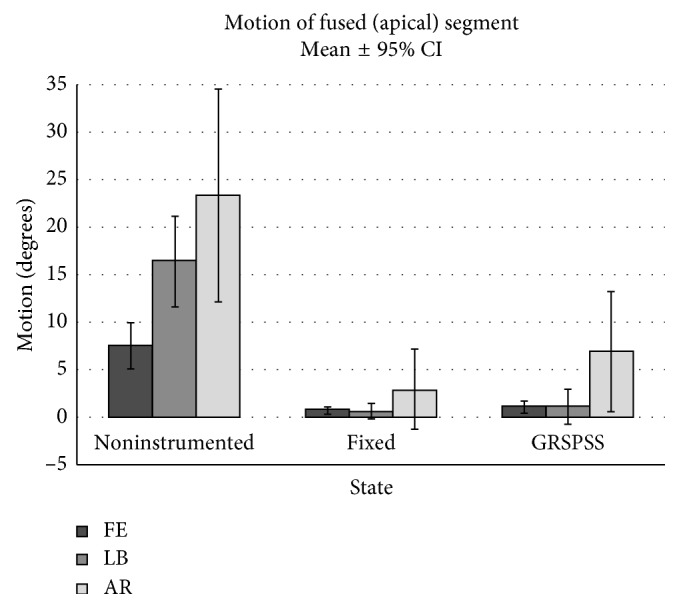
Comparison of apical segment motion in FE, LB, and AR. No significant difference in apical segment motion after fixed or GRSPSS instrumentation. Both fixed and GRSPSS significantly decreased apical segment motion compared to no instrumentation.

The middle T8-10 segment in both the fixed rod and GRSPSS constructs was secured to the rods with conventional pedicle screws with locking set screws, representing the apical segment which is fused during clinical application of growing rod systems [21]. The motion between the T8 and T10 segments represents the stability of the fusion area in both the fixed and GRSPSS constructs (Figure 5). FE segment motion was 0.76° ± 0.42° and 1.05° ± 0.62° for fixed and GRSPSS, respectively (*p*=0.51). Similarly, LB motion was 0.64° ± 0.88° for the fixed state and 1.05° ± 1.86° for the GRSPSS (*p*=0.49). AR motion for the apical segments was significantly different between the fixed rod (2.9° ± 4.2°) and GRSPSS (7.0° ± 6.3, *p*=0.019).

### 3.3. Stiffness

**Figure 6:**
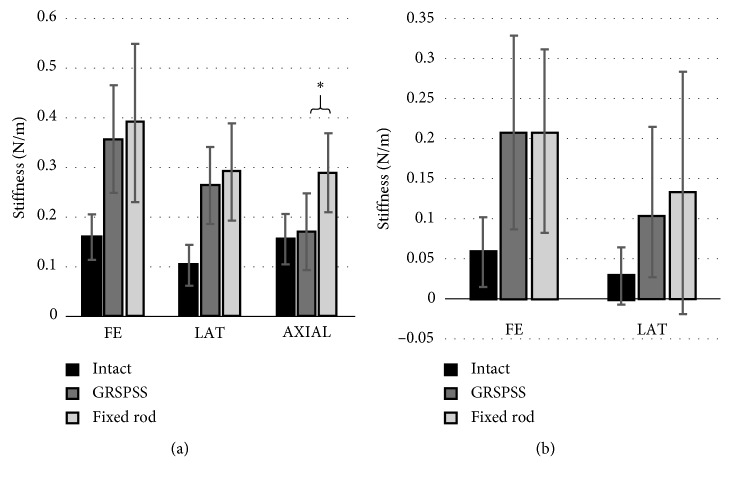
(a) Elastic zone and (b) neutral zone stiffness—with a significance difference found between fixed and GRSPSS in EZ stiffness in AR only. Data are mean ± 95% CI.

Between the GRSPSS and fixed rod states, no significant differences were observed in FE (*p*=0.41) or LB (*p*=0.18) for the EZ stiffness. For AR, the fixed rod EZ stiffness (0.29 N/m ± 0.16 N/m, *p* < 0.001) was significantly greater than the GRSPSS EZ stiffness (0.17 N/m ± 0.08 N/m) (Figure 6). Similarly, for NZ stiffness, no significant differences were observed in FE (*p*=0.96) or LB (*p*=0.31). Due to the high stiffness of the lumbar and thoracic spine in resisting motion and the added stability of the fusion techniques, the AR degree of freedom did not exhibit a detectible NZ region, and therefore, only the EZ stiffness was calculated and reported.

## 4. Discussion

The goal of this study was to evaluate the mechanical stability of a guided growth construct, the GRSPSS system, for use in early-onset scoliosis. The GRSPSS system represented a similar mechanical construct to the FDA-approved SHILLA device. While there has been good clinical follow-up reported for the SHILLA system at two and five years [21, 26], to date, there is little to no *in vitro* biomechanical evidence characterizing the stability of either the GRSPSS or the SHILLA systems. This is particularly concerning given that rod breakage has been reported as a frequent complication associated with the SHILLA system, occurring in up to 30% of patients, and may be in association with increased motion at the fused apical segment [27–29]. In fact, when compared to conventional, fixed rod systems, SHILLA patients ultimately went through almost the same number of surgeries due to complications [27]. It may be that the system's rigidity leads to breakage, but the lack of biomechanical evidence makes it difficult to tell conclusively.

Results of the current study demonstrated that GRSPSS and conventional, fixed rod systems had similar biomechanical stability in FE and LB, but the GRSPSS had less ability to resist overall AR. This finding was further supported by the EZ stiffness data, wherein the fixed rod system had significantly greater EZ stiffness than the GRSPSS in AR. It is hypothesized that the observed decreased stiffness in AR is because the rods are not locked into the sliding screws and therefore are still able to rotate. Despite advantages of the device granted by adding degrees of freedom, GRSPSS may be subject to complications—primarily rods bending or breaking, or screw loosening. Increased AR, which signifies a high degree of motion preservation, may represent difficulty in the device's ability to control rotation at the apex of the curve. However, there are also potential advantages to the decreased stiffness (increased mobility) observed in the GRSPSS. The increased mobility could potentially prevent spontaneous fusion that has been reported in some nonfusion systems [15].

A recent review of SHILLA rod failures noted that, in 27 cases of failure, rods universally broke immediately caudal or cephalad to the fused, apical segment [29]. The current study shows that while guided growth constructs have equivalent sagittal and coronal stability compared with convention fusion techniques, there is increased rotatory instability that is not controlled in either the overall construct or the fused segment due to the ability of the rods to rotate freely within the caudal and cephalad segments. This instability may explain the high rate of rod breakage seen with the SHILLA construct *in vivo*.

One limitation of the current study is the use of straight ovine spines, which may not simulate the biomechanics of pathologic scoliotic spines. McCarthy et al. previously tested the SHILLA system in a straight caprine animal model and showed continued axial growth, averaging 48 mm in six months, without hardware complications or failure [30]. Congenitally scoliotic sheep spines have been created through maternal infections and mentioned in the literature [31], but naturally scoliotic thoracic spines are rare due to quadruped biomechanics. The use of sheep specimens is further justified as an acceptable substitute given the difficulty of obtaining human cadaveric specimens to match the age of the EOS patient population. Another limitation of this study is that it was designed to evaluate the time zero *in vitro* biomechanical properties of the GRSPSS system and therefore, only three cycles of loading were performed. In the clinical setting, the GRSPSS system will be subjected to repetitive loading, and therefore, fatigue testing should also be performed in future work.

This study also does not address the use of crosslinking in growing rod systems and the additional torsional stability that may be offered with their inclusion. McCarthy et al.'s original description of the SHILLA construct (2010) does not include a crosslink in the instrumentation, while radiographic evidence in subsequent clinical studies shows a crosslink [21, 30]. Many biomechanical studies showed that additional crosslinks increase the torsional stiffness of the construct significantly, while other studies demonstrate that crosslinks add very little additional rotational stiffness and may be avoidable in many cases [32–34]. Eliminating crosslinks reduces the operative time as well as the overall cost. Prominence of implants, corrosion, infection, implant failure, and pseudarthrosis are complications attributed to crosslinks in the literature, which can be avoided by preventing their incorporation into spinal constructs [35, 36].

The present study indicated that the GRSPSS performed similarly with the fixed rod system in FE and LB but demonstrated a reduced ability to resist AR. The observation of increased AR in the apical segment represents a potential weakness in controlling apical segment deformity correction and allowing for eventual fusion of this segment. Additionally, this increased motion at the apical segment and within the entire construct may represent a potential causality for the instrumentation failure issues commonly seen in clinical studies of the SHILLA device, which occur around the apical segment. There are many avenues to potentially address the increased rotation motion, including the possibility of using crosslinks. Future research would be needed to evaluate construct stability in rotational motion with the addition of crosslinks and the number of crosslinks needed to obtain similar rotational stiffness to fixed rod systems. Initial biomechanical testing of the GRSPSS system illustrates a potential surgical alternative to conventional fixed rods for treatment of EOS that both maintain movement in the treated spine segment and do not require further surgery either for lengthenings or to address complications.

## Data Availability

The data used to support the findings of this study are available from the corresponding author upon request.
